# Cancer mortality and incidence following external occupational radiation exposure: an update of the 3rd analysis of the UK national registry for radiation workers

**DOI:** 10.1038/s41416-018-0184-9

**Published:** 2018-08-15

**Authors:** Richard G. E. Haylock, Michael Gillies, Nezahat Hunter, Wei Zhang, Mary Phillipson

**Affiliations:** Public Health England, Centre for Radiation, Chemical and Environmental Hazards, Chilton, Didcot, Oxfordshire OX11 0RQ UK

**Keywords:** Cancer epidemiology, Risk factors

## Abstract

**Background:**

This study provides direct evidence of cancer risk from low dose and dose rate occupational external radiation exposures.

**Methods:**

Cancer mortality and incidence were studied in relation to external radiation exposure in the National Registry for Radiation Workers. A cohort of 167,003 workers followed for an average of 32 years was analysed using Poisson regression methods.

**Results:**

Mortality and incidence risks were significantly raised for the group of all malignant neoplasms excluding leukaemia (ERR/Sv mortality = 0.28; 90%CI: 0.06, 0.53, ERR/Sv incidence = 0.28; 90%CI: 0.10, 0.48) but with narrower confidence bounds compared with the previous analysis of this cohort reflecting the increased statistical power from the additional 10 years of follow-up information. The linear trends in relative risk for both mortality and incidence of these cancers remained statistically significantly raised when information relating to cumulative doses above 100 mSv was excluded (ERR/Sv mortality = 1.42; 90%CI: 0.51, 2.38 and ERR/Sv incidence = 1.18; 90%CI: 0.47, 1.92).

**Conclusions:**

This study improved the precision of the cancer risk estimates seen in the third analysis of the NRRW cohort. The overall results remain consistent with the risk estimates from the Life Span Study and those adopted in the current ICRP recommendations.

## Introduction

Much of the evidence that supports radiation health protection standards comes from epidemiological studies of the survivors of the Japanese atomic bombings, primarily the Life Span Study (LSS),^1^ who are estimated to have received a wide range of acute doses of external gamma radiation.^2–4^ Uncertainty remains about how these risk estimates should be extrapolated across populations and from acute to chronic low doses or low dose rate exposures as seen in current occupational exposure or medical diagnostic settings.

The National Registry for Radiation Workers (NRRW) was started in 1976 to provide direct evidence of the risks to health from occupational exposure to chronic low dose external radiation in the UK. The third analysis of the NRRW (NRRW-3)^5,6^ demonstrated a healthy worker effect as seen previously^7–9^ in this and many other occupationally exposed cohorts. It also found statistically significant linear dose–response relationships for mortality from leukaemia (excluding chronic lymphatic) and all cancers excluding leukaemia that agreed well with contemporary estimates from the LSS albeit with considerably wider confidence limits. This analysis includes an additional 10 years of follow-up information (compared to NRRW-3) but does not include additional dosimetry information. Thus the minimum lag period that could be sensibly used for the analysis was 10 years. Therefore updated leukaemia risks are not presented here as leukaemia is usually analysed with a much shorter lag period (e.g. 2 years). The definitions of the disease groups are given in Table S1.

## Materials and methods

### Cohort definition

The background of the NRRW cohort has been reported in detail previously.^9^ Briefly, the cohort consists of individuals monitored for occupational exposure to external ionising radiation, who were employed by participating organisations, and for whom individual dose records were kept. Data collected from employers consisted of individual identifiers, date of birth, sex and industrial classification (industrial or non-industrial where available)—a surrogate for socio-economic status broadly equivalent to manual/non-manual occupations, calendar periods of employment and external radiation dose histories. The three analyses published to date have been based on an audited subset of the overall cohort. The subset used in NRRW-3 and now in this extension has been described before.^5,6^

### Radiation doses

Radiation dose is defined as that from penetrating radiation at the surface of the body and is based on the records from individual’s personal dosimeters. Due to variation in the accuracy and reliability of dosimeters over time corrections are applied to the recorded doses to define a consistent and stable dose estimate for analysis.^9^ The external exposures are predominantly from X-rays and gamma rays but with smaller contributions from beta particles and neutrons. The overall mean 10-year-lagged lifetime external dose was 25.3 mSv. It was higher among the males (27.5 mSv) than the females (5.6 mSv) who constitute just 10% of the cohort. The dose distribution is however very skewed towards low doses. Risk from internal exposure to nuclides such as plutonium, uranium and tritium could not be assessed in detail as only information relating to whether a worker was ever monitored for internal contamination was available for the majority of workers.

### Follow-up

Follow-up data for both mortality and cancer incidence for the cohort has been provided to Public Health England (PHE) by NHS Digital (formerly the Health and Social Care information Centre) and by National Records of Scotland on an ongoing basis. By the end of follow-up on 31 December 2011 over 50% of workers who started radiation work before 1960 had died showing the increased maturity of the cohort (Table S2) and improved statistical power.

### Statistical methods

The mortality analyses were based on the underlying cause of death, coded according to the 9th revision of the International Classification of Diseases (ICD9).^10^ The cancer incidence analyses were based on the first identified cancer (also coded to ICD9) except if that was a non-melanoma skin cancer (NMSC) in which case it was ignored (the analysis of NMSC used all first identified cases). Deaths and cancer incidences that were only reported to NRRW using ICD10^11^ were recoded to ICD9 using general equivalence mapping tables.^12^

The start of follow-up for each worker was taken to be 10 years after the date of start of radiation work with a participating employer, or 10 years following the date on which radiation monitoring data were available, or 1 January 1955, whichever was later. Follow-up prior to 1955 was excluded due to concerns about the reliability of data during this early period of radiation work. Workers were regarded as being at risk until the earliest of their date of death or emigration, their 85th birthday, or 31 December 2011 for the mortality analyses or, for the cancer incidence analyses, until their date of first cancer registration if available (excluding NMSC).

Poisson regression models with a fully stratified baseline hazard function were used. This type of analysis allowed the efficient estimation of the risk of cancer from external radiation having taken into account available non-radiation factors known to modify the baseline cancer risk. In the analyses the person-years and deaths (or cancer incidences) were stratified by non-radiation factors age (in 5-year groups), sex, calendar period (1955–, 1960–,…, 2010–2011), industrial classification (industrial/non-industrial/unknown) and 15 first employer groups^5^^,6^ and were also classified by cumulative external dose in the categories 0–, 10–, 20–, 50–, 100–, 200–, 400 + mSv. Doses were lagged 10 years to define the minimum period between exposure and the first possible detection of radiation induced disease.

Sensitivity analyses were conducted to examine the impact on the results of deaths and cancers occurring at ages over 85, duration of radiation work and by whether or not workers had ever been monitored for exposure to internal emitters.

Point estimates of the excess relative risk (ERR) per sievert were estimated by fitting linear models and maximising the associated likelihood function using the same in-house Fortran based software as used previously to ensure backward comparability of results. Only strata in which person-years and at least one death accrued in more than one cumulative dose group were informative. The statistical significance of each model and the confidence intervals (CI) for the ERR were derived using the score statistic.^13,14^ Since there was a prior hypothesis of a positive association of cancer rates with increasing external radiation dose one-sided *p*-values and corresponding 90%CI are reported in the text (both one and two-sided *p*-values are given in the supplementary tables). For models with <100 informative strata the *p*-values were based on simulation. Risks were estimated for the same set of solid cancer groups (excluding leukaemia) as used in the NRRW-3 analysis with the additional grouping of all solid cancers, which was not used in NRRW-3.

## Results

Table 1 shows the characteristics of the cohort.

**Table 1:** Characteristics of cohort members who contribute to the 10-year-lagged mortality analysis

	Overall^a^	Male	Female
Population	167,003	150,566 (90%)	16,437 (10%)
Industrial	94,432 (56%)	87,998 (58%)	6,434 (39%)
Non-industrial	70,322 (42%)	60,889 (40%)	9,433 (57%)
Unknown	2,249 (2%)	1,679 (2%)	570 (4%)
Monitored for internal exposure	24.6%	25.5%	17.3%
Deceased with cause of death	34,955 (20.9%)	33,626 (22.3%)	1,329 (8%)
Person-years	3,684,391	3,363,053	321,338
Mean length of follow-up^b^	32 years 3 months	32 years 6 months	29 years 8 months
Mean age at start of follow-up	29 years 8 months	30 years	27 years 2 months
Mean age at last observation^c^	61 years 9 months	62 years 4 months	56 years 8 months
Mean dose^d^	25.3 mSv	27.5 mSv	5.6 mSv
Median dose^d^	3 mSv	3.5 mSv	1.2 mSv
	Monitored for internal exposures	Not monitored for internal exposures	
Population proportion	25%	75%	
Mean dose^d^	62.1 mSv	13.3 mSv	
Median dose^d^	17.1 mSv	1.6 mSv	

^a^Numbers quoted here relate only to workers who contribute to the 10-year-lagged analysis but those in the 3rd analysis publications relate to the whole cohort.^5,6^

^b^The figure includes the first 10 years of follow-up which are excluded from the analysis.

^c^Assumes end of follow-up at 85th birthday for those still alive at that age.

^d^10-year-lagged dose

### Mortality

**Fig. 1:**
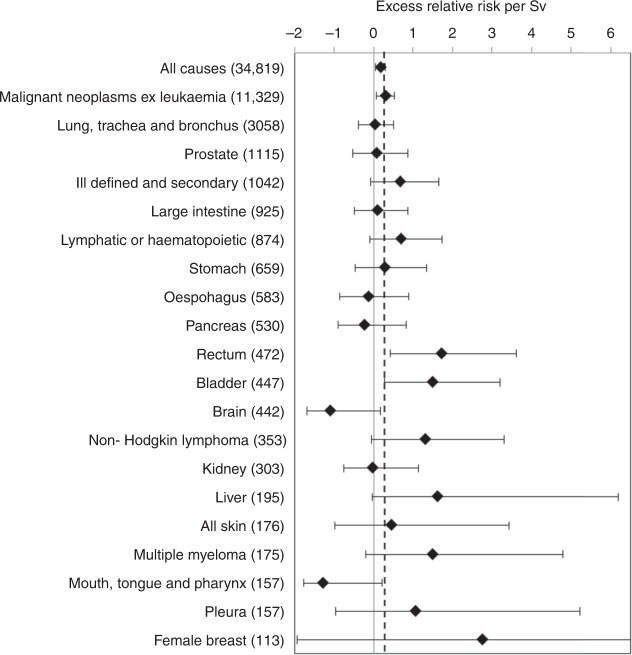
Point estimate and 90%CI of the linear trend in excess relative risk per sievert of death with 10-year-lagged dose for summary groupings and cancer sites with the greatest number of deaths (in parentheses). The dotted vertical line indicates the risk for malignant neoplasms excluding leukaemia

The point estimates of ERR per Sv and associated 90%CI’s for the summary disease groups and the 19 cancer sites with the greatest number of informative deaths are shown in Fig. 1. The full results are provided in the supplementary Table S3.

**Fig. 2:**
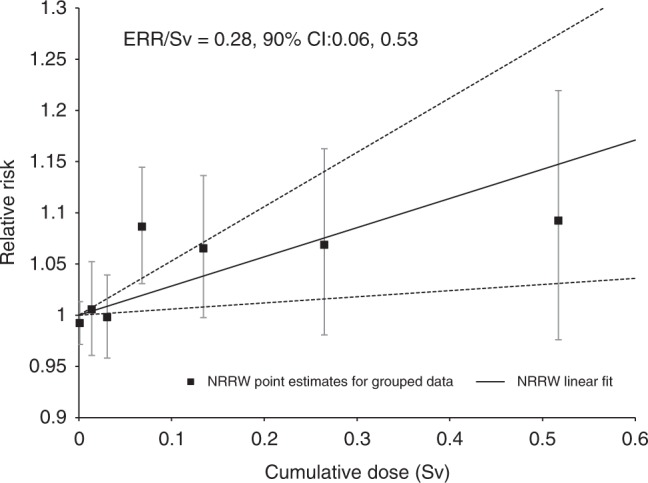
Trend in ERR with dose (and 90% CI) for mortality from all malignant neoplasms excluding leukaemia

There was strong evidence of a positive association between all-cause mortality risk and 10-year-lagged cumulative dose (ERR/Sv = 0.17; 90%CI 0.05, 0.30; *p* = 0.009) based on 34,819 deaths. Figure 2 illustrates the increasing trend in mortality with dose for the group of all malignant neoplasms except leukaemia (ERR/Sv = 0.28; 90%CI 0.06, 0.53; *p* = 0.02, 11,329 deaths). Also plotted for each dose group is an estimate of relative risk (the ratio of the observed number of deaths within each cumulative dose group over the number of deaths that would be expected if there were no association between the risk of mortality and dose) and Poisson-based 90% confidence interval. The additional exclusion of lung and pleural cancers from this summary group, cancers which are highly likely to be caused by smoking or asbestos exposure, caused the slope of the trend to increase (ERR/Sv = 0.37, 90%CI 0.11, 0.65; *p* = 0.009, 8114 deaths) although the precision of the estimate decreased.

The point estimates of risk among the major individual cancer groups (Fig. 1) were positive except for oesophageal, pancreatic, brain, kidney and cancers of the tongue, mouth and pharynx. Significantly raised ERR estimates were observed for cancers of the rectum (ERR/Sv = 1.72; 90%CI 0.42, 3.61; *p* = 0.009, 472 deaths) and bladder (ERR/Sv = 1.49; 90%CI 0.28, 3.19; *p* = 0.017, 447 deaths) and there was limited evidence of raised risk for liver cancer and non-Hodgkin’s lymphoma (*p* = 0.051 and 0.058, respectively). For each of the main sub-types of solid cancer there was no evidence of deviation of risk estimates from the overall solid cancer risks estimate and no overall evidence of heterogeneity of risk across the cancer sites (*p* = 0.48).

The result of the sex-specific analyses revealed very similar results for males to the main analysis although the *p*-value for liver cancer became statistically significant (ERR/Sv = 2.55; 90%CI 0.04, 6.58; *p* = 0.046). Female workers generally had smaller exposures and a total of only 1255 deaths were observed, as a consequence, the female-specific analysis lacked the statistical power to be informative. The results of the analyses that included 3861 deaths which occurred at attained ages over 85, of which 763 were malignant solid cancers, were virtually unchanged from the main analysis.

The influence of internally monitored workers was examined for the main disease groupings in two ways. The first method included an additional stratification in the risk model for monitoring status while the second method excluded workers monitored for internal emitters.

The results using the first method resulted in the loss of the statistical significance of the raised risk estimate for all malignant neoplasms excluding leukaemia but the result when lung and pleural cancer were additionally excluded was unchanged. Other results also remained broadly the same. Excluding the monitored workers reduced the number of cancer deaths by 31%. The risk estimate for all malignant neoplasms excluding leukaemia increased (ERR/Sv = 0.66; 90%CI 0.20, 1.18; *p* = 0.008; 7820 deaths) as did the estimate when lung and pleural cancers were also excluded (ERR/Sv = 0.74; 90%CI 0.22, 1.33; *p* = 0.008; 5685 deaths). These estimates were consistent with those for the overall cohort although less precise. The risk estimates for cancers of the rectum and bladder both lost statistical significance while that for stomach cancer increased by almost a factor of 10 (ERR/Sv = 2.65; 90%CI 0.60, 5.82; *p* = 0.01; 431 deaths).

**Fig. 3:**
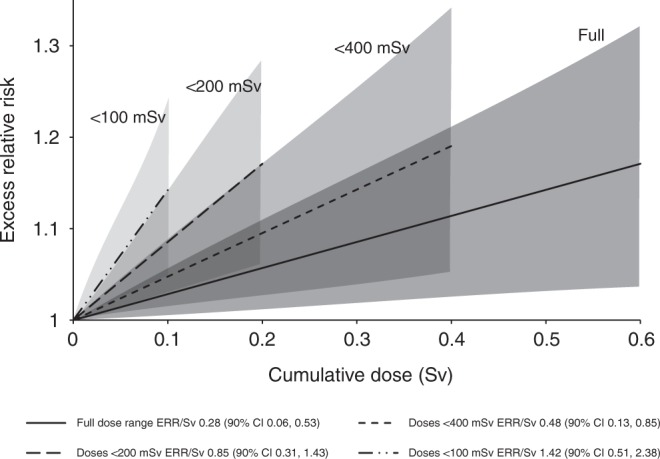
Trends in ERR with dose (and 90% CI) for mortality from all malignant neoplasms excluding leukaemia with restricted cumulative doses

To assess the strength of the dependence of the overall trend for malignant neoplasms excluding leukaemia on the more highly exposed workers the analysis of this overall grouping was repeated excluding person-years experience accrued at cumulative doses exceeding 400, 200 and 100 mSv respectively. The excess risk remained significantly raised down to maximum doses of 100 mSv (ERR/Sv = 1.42; 90%CI 0.51, 2.38; *p* = 0.005; 10,068 deaths) (Fig. 3) but when lung and pleural cancers were additionally excluded from the group a significant excess risk was only seen when using the full dose range.

The influence of longer lag periods of 15 and 20 years on the result for malignant neoplasms excluding leukaemia was examined. The 15-year-lag result was very similar to the standard 10-year-lag result while the 20-year-lag risk value was a little higher (Lag 15: ERR/Sv = 0.27 90%CI 0.03, 0.53; *p* = 0.03, Lag 20: ERR/Sv = 0.35; 90%CI 0.08, 0.65; *p* = 0.01).

An analysis incorporating an extra baseline stratum for duration of exposure with three levels (0–9, 10–29 and 30+ years) was performed. The all-cause risk result was very similar. The result for all malignant neoplasms excluding leukaemia also remained significantly raised (ERR/Sv = 0.37; 90%CI 0.09, 0.88; *p* = 0.01). The raised ERR estimates for bladder and rectal cancer were still present but the significance of the later result was reduced (*p* = 0.051).

### Incidence

**Fig. 4:**
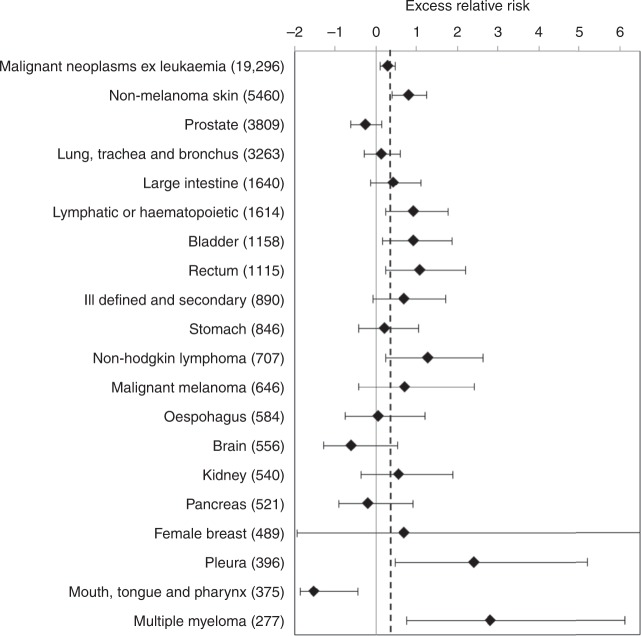
Point estimate and 90%CI of the linear trend in excess relative risk per Sv of cancer incidence with 10-year-lagged dose: summary groupings and cancer sites with the greatest number of cases (in parentheses). The dotted vertical line indicates the risk for malignant neoplasms excluding leukaemia

Figure 4 shows the ERR per Sv point estimates and 90%CI for all malignant neoplasms excluding leukaemia and the 19 cancer sites with the greatest number of informative cases. The full results are provided in the supplementary Table S4. Overall the number of informative malignant cancer incidences excluding leukaemia and NMSC was 19,296.

The point estimate of the risk for all malignant neoplasms excluding leukaemia and NMSC was marginally higher (ERR/Sv = 0.28; 90%CI 0.10, 0.48; *P* = 0.005, 19,296 cancers) and with a narrower confidence interval than in the NRRW-3 analysis. Among the individual cancer sites in Fig. 4 all of the point estimates of risk were positive except for cancers of prostate, brain, pancreas and the grouping of mouth, tongue, and pharyngeal cancers. The last grouping was the only one for which the 90% and 95% upper confidence limits were below one) but when the single case above 400 mSv was excluded there was no evidence that the negative risk estimate was statistically different from zero. Among the site-specific risk estimates those for, bladder, multiple myeloma, NMSC, non-Hodgkin’s lymphoma, rectum and all lymphatic and haematopoietic cancer were significantly raised.

The same range of sensitivity analyses as performed on the mortality data were undertaken. There were still insufficient female cases for an informative analysis of sex effects while the male-specific results were unchanged from the main analysis as were those when including cases occurring over 85 years of age. In contrast to the mortality analysis adding an extra stratification for monitoring for internal exposure had no effect on the result for malignant neoplasms excluding leukaemia but did show a significantly raised risk estimate for cancer of the large intestine (ERR/Sv = 0.71; 90%CI 0.06, 1.55; *p* = 0.034, 1636 cases) and a loss of statistical significance in the risk estimate for non-Hodgkin’s lymphoma. As with the mortality results, excluding those workers monitored for internal emitters resulted in higher point estimates of excess risk for all malignant neoplasms excluding leukaemia (ERR/Sv = 0.62; 90%CI 0.25, 1.02; *p* = 0.002; 13,985 cases) though with much lower precise than when using the whole cohort. Among the site-specific results in Fig. 4 only the risk estimate for rectal cancer remained significantly raised in the reduced cohort. Adding stratification for duration of exposure to the incidence analyses increased the point estimate of the ERR/Sv for malignant neoplasms excluding leukaemia to 0.34 (*p* = 0.006) but when lung and pleural cancers were also excluded the extra stratification had no effect on the estimate. Among the individual sites the raised risk for lymphatic and haematopoietic cancer lost significance.

## Discussion

These analyses were based on the NRRW-3 cohort (with minor corrections) with follow-up extended by 10 years and include 34,819 informative deaths among 3.68 million person-years a large increase over the 23,326 deaths and 2.43 million person-years seen in the previous 10 year lagged mortality analysis.

### General patterns of mortality and cancer incidence

The estimates of the excess risk of death and incidence from all malignant neoplasms excluding leukaemia (and NMSC) remained statistically significantly raised while the span of associated the 90% confidence bounds were narrower. In both instances the strength of support for these not being chance findings (*p* = 0.017 and *p* = 0.005, respectively) increased compared to NRRW-3. The additional exclusion of lung and pleural cancer had little impact on the results and an analysis of chronic obstructive pulmonary disease (COPD) did not show any positive association of risk with dose (Table S3) although it did show some evidence of a negative association. These results taken together with the lung cancer estimates suggest that smoking is unlikely to be a positive potential confounder when examining external radiation effects. If anything the low estimates for COPD and lung cancer may point towards some negative confounding effect of smoking on radiation estimates. However, non-cancer diseases also associated with smoking (although less strongly so) have shown positive associations with radiation exposure in prior analysis of this cohort so the potential confounding effects of smoking may be not be open to any simple interpretation.

Restricting analyses to data regarding doses below 400, 200 and 100 mSv indicated that the raised risk estimates per unit dose at low doses were not reliant solely on the data in the high dose categories. The attenuation of risk per unit dose at higher cumulative doses may in part be the result of a selection process with workers who remain employed for long periods and thus accumulate higher doses tend to be healthier than those who leave employment (the ‘healthy worker survivor effect’ (HWSE)).^15^ Although the HWSE may play a role in the pattern of results observed an adjustment for duration of employment did not materially affect the risk estimates and it may be that selection into internal radiation work (the majority of high dose workers were also internally monitored) results in a selection effect beyond the normal HWSE.

The impact of exposure to internal emitters on the estimates of risk from external exposure was assessed by incorporating an additional baseline stratum into the model but this did not materially affect the overall risks. However, excluding internally monitored workers from the analysis resulted in a doubling of the point estimate of mortality risk and an increase of 60% of the incidence risk for all malignant neoplasms excluding leukaemia. In both instances the width of the 90% confidence interval was also doubled. Overall 24% of the workers analysed were monitored for potential internal exposures but this group, who tended to receive higher external doses, account for 67% of the overall cancer deaths amongst workers who received doses in excess of 100 mSv. Below 100 mSv the results for the monitored and non-monitored groups were nearly identical with an ERR/Sv of 1.2. Thus attenuation in risk is happening for both groups when including higher doses but the effect is stronger for monitored workers perhaps suggesting the HWSE or a selection effect into continued employment or internal work. All NRRW analyses to date have used a fixed internal monitoring factor that assumed monitoring starts from the beginning of radiation work. This is not ideal as some workers will not have been monitored for internal exposure from first employment so their early person-years will be misclassified but the additional information to construct a time varying factor is currently not available for the cohort. However a time varying factor was used for the recent BNFL cohort analysis^16^ and this showed the same pattern of risk but further investigation of this issue is needed.

### Comparison with other studies

There was good agreement of the risks for both mortality and incidence of solid cancers and solid cancers excluding lung and pleural cancer from this study and risks derived for this study by fitting a linear model to a comparable subset of the LSS14 data^17,18^ (Table 2). The largest difference was for solid cancer mortality where the updated NRRW result was 17% smaller than the comparable LSS14 based estimate. Comparing this study with the International Workers Study (INWORKS),^19^ which incorporated the 85% of NRRW-3 cohort who had nuclear industry employers, the INWORKS risk estimate for all solid cancer was 37% higher. Excluding lung cancer reduced this difference only slightly. Given the span of the confidence limits these results were still in broad agreement.

**Table 2:** Comparison of estimates of linear ERR per Sv (and 90% CI) with other studies

	Solid cancer	Solid cancer excluding lung and pleura cancer
Updated 3rd NRRW analysis		
Mortality	0.24 (0.01, 0.48)	0.30 (0.04, 0.60)
Incidence (excludes non-melanoma skin cancer)	0.22 (0.03,0.42)	0.20 (−0.01, 0.42)
3rd NRRW analysis^5,6^		
Mortality^a^	0.20 (−0.08, 0.50)	0.25 (−0.08, 0.62)
Incidence^a^ (excludes non-melanoma skin cancer)	0.21 (−0.02, 0.46)	0.19 (−0.06, 0.48)
INWORKS mortality^19^	0.33 (0.12, 0.56)^b^	0.32 (0.07, 0.60)^c^
Japanese atomic bomb survivor data (males exposed to 20–60, <2 Gy, linear model)		
Mortality^d^	0.29 (0.17; 0.43)	0.27 (0.13, 0.42)
Incidence^e^	0.30 (0.16; 0.46)	0.33 (0.17; 0.52)
Mayak worker cohort (1948–82)		
Mortality		0.12 (95% CI 0.03, 0.21)^f^
Incidence	n/a	0.06 (90% CI −0.001, 0.13) ^g^

^a^Based on the underlying cause figures were not previously published.

^b^Result for solid cancer excluding leukaemia based on recorded external dose.

^c^Result for solid cancer excluding lung based on recorded external dose.

^d^Males exposed between 20 and 60 years of age to < 2Gy colon dose, linear model.^17^

^e^Incidence results are based on solid cancers only; males exposed between 20 and 60 years of age to < 2Gy colon dose, linear model.^23^

^f^Male result for solid cancers excluding lung, liver and bone based on a linear model for external dose with adjustment for plutonium exposure.^20^

^g^Gender-averaged value^21^

The risk estimates for cancer mortality and incidence from the Mayak worker cohort^20,21^ are lower than the other risk estimates in Table 2. The discordance between the Mayak risks and those of the LSS and other occupational studies has been noted previously.

### Individual cancer sites

The significantly raised risks for mortality and incidence of rectal cancer found here were seen in NRRW-3 but those for bladder cancer were not. In the LSS mortality analysis^17^ the overall risk for rectal cancer was not significantly raised nor was it for males but for females the risk was raised (ERR/Gy = 0.66; 95%CI 0.06; 1.5; *p* = 0.03, 228 deaths). Unfortunately, in this study the female-specific analysis of rectal cancer was uninformative as there were only 14 deaths of which eight were in the lowest dose category. For bladder cancer mortality the overall and sex-specific LSS mortality risks were all significantly raised with that for females having almost twice the risk of males. Here only nine bladder cancers were seen among females and the main mortality result which is almost a male-specific result was close to the LSS14 result for females. This difference may be explained by variation in underlying rates of disease between males and females and countries rather than a real difference in radiation risks.

For ovarian cancer, lymphatic and haematopoietic cancer, pleural cancer, non-Hodgkin’s lymphoma and multiple myeloma significantly raised risks were seen in this study for incidence but not mortality. Only for the latter two diseases were there comparable raised risks seen in the NRRW-3 analysis. The ovarian cancer result derived here was based on 61 cases but only two occurred in workers with doses of 50 mSv or more so is not very reliable. Incidence risks derived from the LSS^18,22^ for all these cancer groupings (except pleural cancer), provided either weak or no evidence of raised risk. The site-specific analyses restricted to workers who were not monitored for internal exposure revealed that only for rectal cancer incidence was there a statistically significant excess risk (ERR/Sv = 1.70; 90%CI 0.16, 3.94; *p* = 0.031; 769 cases) which was higher than the overall rectal cancer result.

### Limitations of the study

The biggest source of uncertainty likely relates to the dosimetry. In particular early dosimeters (film badges) had high threshold detection limits and the assessment of neutron exposure particularly in the first 20–30 years of follow-up was poor. Attempts to take account of neutron exposure information in the recent INWORKS^19^ project resulted in a variation of the solid cancer risk estimate of a factor of 2 from the primary result. A further drawback was the lack of quantitative internal dose estimates, the use of an indicator in the model stratification revealed issues that need further consideration. Although this study is a predominately a low dose and dose rate study earlier workers who accrued larger exposures (>100 mSv) over a number years do have a significant leverage on overall risks estimates but significant risks were still observed when restricting the analysis to low doses (<100 mSv) allbeit with greater uncertainty. Finally, potentially confounding lifestyle factors such as smoking, hypertension and BMI were not available.

### Future work

In further work the shape of dose–response will be examined in detail as will the temporal variation in radiation risk (mortality and cancer incidence) with age and time since exposure. A analysis on the potential association between non-cancer disease mortality risk and radiation exposure is already underway.

## Conclusions

This analysis confirms the association seen between cancer risks and occupational external radiation exposure seen in NRRW-3. The estimates observed here are more precise than those reported previously and remain consistent with those derived from the published Life Span Study data.

## Electronic supplementary material

Supplementary material
