# Supplementary material for: Cancer mortality and incidence following external occupational radiation exposure: an update of the 3rd analysis of the UK national registry for radiation workers
Source: Br J Cancer. 2018 Aug 15;119(5):631–7. doi: 10.1038/s41416-018-0184-9 (PMC6162263; doi:10.1038/s41416-018-0184-9)
Supplement: Supplementary file 1 — Supplementary material [file 41416_2018_184_MOESM1_ESM.docx]

**Supplementary material**

| **Table S1: definitions of the disease categories** | |
| --- | --- |
| **Definition of disease groupings** | **ICD 9th revision codes** |
| All causes | 000-999 |
| All neoplasms | 140-239 |
| All known causes excluding malignant neoplasms | 000-139 209-799.8 800-999.8 |
| All malignant neoplasms excluding leukaemia | 140-202.3, 202.5-203, 203.2-203.9 |
| All malignant neoplasms excluding leukaemia, lung and pleura | 140-161.9, 164-202.3, 202.5-203, 203.2-203.9 |
| All solid cancers | 140-199 |
|  |  |
| **Specific Malignancies** |  |
| Mouth, tongue and pharynx | 141, 143-148, 149.0 |
| Oesophagus | 150 |
| Stomach | 151 |
| Large intestine | 153, 159.0 |
| Rectum | 154.0-154.2 154.4-154.9 |
| Liver | 155 |
| Primary liver | 155.0 |
| Gall bladder | 156 |
| Pancreas | 157 |
| Larynx | 161 |
| Trachea, bronchus and lung | 162 |
| Pleura | 163 |
| Bone | 170 |
| Connective and soft tissue | 171 |
| All skin | 172-173 |
| Non melanoma skin cancer | 173 |
| Female breast | 174 |
| Uterus | 179-182 |
| Ovary | 183 |
| Prostate | 185 |
| Testis | 186 |
| Bladder | 188, 189.3-189.9 |
| Kidney | 189.0-189.2 |
| Brain | 191-192, 224-225, 239.6 |
| Thyroid | 193 |
| Ill-defined and secondary cancers | 195-199 |
| All lymphatic and haematopoietic | 200-208, 238.6 |
| Non-Hodgkin lymphoma | 200, 202.2-202.3, 202.5-202.9 |
| Hodgkin lymphoma | 201 |
| Multiple myeloma | 203.0, 203.2-203.9, 238.6 |
| Malignant neoplasms strongly related to smoking | 141, 143.0-149.0, 150.0-150.9, 157.0-157.9, 161.0-163.9, 188.0-189.9 |

| **TABLE S2: All cause mortality and percentage of the population deceased by 01/01/2012.^1^** | | | | | |
| --- | --- | --- | --- | --- | --- |
|  | Cumulative 10 year lagged dose (mSv) | | | | |
| Calendar period of first radiation work | <10 | 10 - 49 | 50 - 99 | 100+ | Total |
| <1955 | 1762 (64%) | 2229 (64%) | 949 (64%) | 1437 (68%) | 6377 |
| 1955 - 1959 | 2196 (48%) | 2280 (51%) | 790 (53%) | 1328 (59%) | 6594 |
| 1960 - 1969 | 7333 (33%) | 3358 (38%) | 1019 (40%) | 1181 (43%) | 12891 |
| 1970 - 1979 | 4234 (15%) | 1442 (16%) | 426 (16%) | 441 (17%) | 6543 |
| 1980 - 1989 | 1758 (6%) | 346 (5%) | 73 (6%) | 49 (9%) | 2226 |
| 1990 – 2001 | 307 (1%) | 16 (2%) | 1 (1%) | Category empty | 324 |
| Total | 17590 | 9671 | 3258 | 4436 | 34955 |

**^1^** results based on the overall population and number of deaths in the cohort as defined in Table 1

| **TABLE S3 Test for trend in mortality with dose by cause of death (lagged by 10 years)** | | | | | | | | | | | | | | | | | |  |
| --- | --- | --- | --- | --- | --- | --- | --- | --- | --- | --- | --- | --- | --- | --- | --- | --- | --- | --- |
| Dose (mSv) | *Number of deaths* | All Causes | All known causes excluding malignant neoplasms | | All neoplasms | | All malignant neoplasms | All solid cancers | | All malignant neoplasms excluding leukaemia | All malignant neoplasms excluding lung, pleura and leukaemia | | Mouth, tongue and pharynx | Oesophagus | | Stomach | | |
| <10 | *Obs­^1^* | 18384 | 11758 | | 6357 | | 6283 | | 5810 | 6100 | 4413 | | 101 | 341 | | 312 | | |
|  | *Exp^2^* | 18481.95 | 11806.76 | | 6423.23 | | 6355.7 | | 5884.92 | 6186.14 | 4455.69 | | 97.27 | 334.36 | | 327.88 | | |
| 10- | *Obs* | 4167 | 2765 | 1360 | | 1346 | | | 1244 | 1316 | 964 | 24 | | 69 | 83 | | |  |
|  | *Exp* | 4175.31 | 2758.52 | 1376.49 | | 1359.81 | | | 1254.06 | 1322.18 | 944.31 | 15.92 | | 64.31 | 80.73 | | |  |
| 20- | *Obs* | 5225 | 3463 | 1717 | | 1694 | | | 1573 | 1650 | 1149 | 14 | | 79 | 108 | | |  |
|  | *Exp* | 5316.06 | 3522.86 | 1741.53 | | 1721.71 | | | 1593.45 | 1674.75 | 1193.4 | 19.41 | | 81.22 | 104.91 | | |  |
| 50- | *Obs* | 3050 | 2000 | 1031 | | 1024 | | | 959 | 1002 | 694 | 9 | | 38 | 74 | | |  |
|  | *Exp* | 2975.66 | 1971.94 | 974.72 | | 963.77 | | | 894.55 | 937.61 | 668.88 | 10.83 | | 45.49 | 60.66 | | |  |
| 100- | *Obs* | 2027 | 1339 | 677 | | 669 | | | 616 | 651 | 454 | 4 | | 24 | 36 | | |  |
|  | *Exp* | 1989.76 | 1322.74 | 648.62 | | 641.2 | | | 594.95 | 624.53 | 444.17 | 7.2 | | 30.14 | 41.57 | | |  |
| 200- | *Obs* | 1261 | 853 | 396 | | 394 | | | 365 | 384 | 269 | 4 | | 23 | 29 | | |  |
|  | *Exp* | 1190.03 | 796.89 | 383.42 | | 379.13 | | | 352.59 | 369.62 | 257.58 | 4.29 | | 17.14 | 26.78 | | |  |
| 400+ | *Obs* | 705 | 465 | 232 | | 231 | | | 212 | 226 | 171 | 1 | | 9 | 17 | | |  |
|  | *Exp* | 690.22 | 463.3 | 221.99 | | 219.69 | | | 204.49 | 214.17 | 149.98 | 2.07 | | 10.34 | 16.46 | | |  |
| Total deaths in informative strata |  | 34819 | 22643 | 11770 | | 11641 | | | 10779 | 11329 | 8114 | 157 | | 583 | 659 | | |  |
| Score statistic |  | 2.36 | 1.56 | 1.86 | | 2.05 | | | 1.74 | 2.13 | 2.36 | -1.48 | | -0.27 | 0.53 | | |  |
| 1-sided p-value |  | 0.009 | 0.06 | 0.031 | | 0.02 | | | 0.041 | 0.017 | 0.009 | 0.93 | | 0.606 | 0.296 | | |  |
| 2-sided p-value |  | 0.018 | 0.119 | 0.063 | | 0.041 | | | 0.082 | 0.033 | 0.018 | 0.14 | | 0.788 | 0.593 | | |  |
| ERR Sv^-1^ |  | 0.173 | 0.14 | 0.243 | | 0.27 | | | 0.238 | 0.285 | 0.366 | -1.285 | | -0.146 | 0.274 | | |  |
| 90% CI |  | (0.05, 0.30) | (-0.01, 0.30) | (0.03, 0.48) | | (0.05, 0.51) | | | (0.01, 0.48) | (0.06, 0.53) | (0.11, 0.65) | (-1.77, 0.21 ) | | (-0.86, 0.89) | (-0.47, 1.34) | | |  |
| 95% CI |  | (0.03, 0.33) | (-0.04, 0.33) | (-0.01, 0.53) | | (0.01, 0.56) | | | (-0.03, 0.53) | (0.02, 0.58) | (0.06, 0.71) | (-1.80, 0.65 ) | | (-0.97, 1.13) | (-0.59, 1.57) | | |  |

^1^ the observed number of informative deaths in each cumulative dose category

^2^ the expected number of deaths in each dose category if there were no association between mortality and cumulative dose.

| **TABLE S3 (cont.) Test for trend in mortality with dose by cause of death (lagged by 10 years)** | | | | | | | | | | | |  |
| --- | --- | --- | --- | --- | --- | --- | --- | --- | --- | --- | --- | --- |
| Dose (mSv) | *Number of deaths* | Large intestine | Rectum | Liver | Primary Liver | Gallbladder | Pancreas | Larynx | Trachea, bronchus and lung | Pleura | Bone | |
| <10 | *Obs* | 500 | 233 | 110 | 55 | 25 | 288 | 50 | 1609 | 78 | 6 | |
|  | *Exp* | 494.13 | 255.27 | 112.55 | 53.75 | 21.61 | 288.54 | 52.63 | 1645.32 | 85.13 | 6.49 | |
| 10- | *Obs* | 112 | 59 | 18 | 5 | 5 | 65 | 13 | 339 | 13 | 2 | |
|  | *Exp* | 108.88 | 54.21 | 23 | 10.9 | 4.98 | 62.85 | 10.69 | 361.12 | 16.76 | 1.73 | |
| 20- | *Obs* | 123 | 73 | 29 | 15 | 7 | 77 | 13 | 473 | 28 | 2 | |
|  | *Exp* | 139.59 | 69.8 | 27.29 | 13.08 | 6.7 | 78.7 | 12.87 | 458.9 | 22.45 | 1.79 | |
| 50- | *Obs* | 93 | 51 | 13 | 9 | 1 | 47 | 4 | 295 | 13 | 2 | |
|  | *Exp* | 79.19 | 41.17 | 15.24 | 7.39 | 3.95 | 43.57 | 7.05 | 255.32 | 13.42 | 0.9 | |
| 100- | *Obs* | 50 | 23 | 14 | 5 | 2 | 30 | 5 | 182 | 15 | 0 | |
|  | *Exp* | 54.59 | 27.07 | 9.71 | 4.87 | 2.46 | 29.18 | 5.06 | 170.79 | 9.57 | 0.5 | |
| 200- | *Obs* | 25 | 19 | 8 | 4 | 1 | 13 | 4 | 108 | 7 | 0 | |
|  | *Exp* | 30.75 | 15.76 | 4.6 | 2.54 | 1.51 | 17.36 | 2.94 | 106.15 | 5.88 | 0.4 | |
| 400+ | *Obs* | 22 | 14 | 3 | 1 | 1 | 10 | 4 | 52 | 3 | 0 | |
|  | *Exp* | 17.87 | 8.73 | 2.62 | 1.48 | 0.79 | 9.8 | 1.76 | 60.39 | 3.8 | 0.19 | |
| Total deaths in informative Strata |  | 925 | 472 | 195 | 94 | 42 | 530 | 93 | 3058 | 157 | 12 | |
| Score statistic |  | 0.21 | 2.36 | 1.61 | 0.35 | -0.56 | -0.42 | 1.76 | 0.11 | 0.68 | -0.82 | |
| 1-sided p-value |  | 0.418 | 0.009 | 0.054 | 0.341 | 0.693 | 0.664 | 0.051 | 0.458 | 0.248 | 0.771 | |
| 2-sided p-value |  | 0.836 | 0.018 | 0.108 | 0.681 | 0.615 | 0.672 | 0.101 | 0.916 | 0.497 | 0.457 | |
| ERR Sv^-1^ |  | 0.083 | 1.716 | 1.61 | 0.69 | -0.694 | -0.233 | 2.588 | 0.028 | 1.057 | <-1.933 | |
| 90% CI |  | (-0.49, 0.87) | (0.42, 3.61) | (-0.04, 6.18 ) | (-1.48, 5.79) | (-1.58, 2.33) | (-0.91, 0.83) | (0.11, 7.63) | (-0.38, 0.51) | (-0.96, 5.21) | (<-1.93, 25.3) | |
| 95% CI |  | (-0.58, 1.05) | (0.22, 4.04) | (-0.37, 7.08 ) | (-1.61, 7.19) | (-1.65, 3.30) | (-1.00, 1.10) | (-0.18, 9.06) | (-0.44, 0.63) | (-1.17, 6.29) | (<-1.93, 44.8) | |

| **TABLE S3 (cont.) Tests for trend in mortality with dose by cause of death (lagged by 10 years)** | | | | | | | | | | | |  |  |
| --- | --- | --- | --- | --- | --- | --- | --- | --- | --- | --- | --- | --- | --- |
| Dose (mSv) | | *Number of deaths* | Connective and soft tissue | All skin | Female breast | Uterus | Ovary | Prostate | Testis | Bladder | Kidney | All Brain | |
| <10 | | *Obs* | 33 | 110 | 88 | 24 | 30 | 569 | 8 | 215 | 172 | 269 | |
|  | | *Exp* | 30.83 | 107.72 | 89.29 | 22.71 | 32.8 | 579.11 | 8.78 | 225.02 | 167.04 | 267.99 | |
| 10- | | *Obs* | 6 | 13 | 13 | 3 | 4 | 132 | 1 | 58 | 40 | 55 | |
|  | | *Exp* | 5.17 | 17.68 | 12.35 | 3.65 | 4.02 | 131.91 | 1.3 | 54.51 | 34.89 | 50.73 | |
| 20- | | *Obs* | 3 | 21 | 7 | 1 | 6 | 186 | 1 | 68 | 41 | 59 | |
|  | | *Exp* | 5.69 | 22.51 | 7.89 | 2.72 | 3.16 | 175.73 | 1.43 | 71.62 | 44.72 | 56.92 | |
| 50- | | *Obs* | 5 | 15 | 4 | 1 | 1 | 95 | 1 | 38 | 26 | 26 | |
|  | | *Exp* | 3.49 | 12.5 | 2.12 | 0.62 | 0.62 | 101.81 | 0.74 | 40.03 | 24.63 | 30.92 | |
| 100- | | *Obs* | 2 | 10 | 0 | 0 | 0 | 70 | 1 | 29 | 9 | 21 | |
|  | | *Exp* | 2.19 | 8.21 | 1.08 | 0.23 | 0.31 | 66.38 | 0.43 | 27.88 | 16.48 | 18.98 | |
| 200- | | *Obs* | 0 | 4 | 1 | 1 | 0 | 43 | 1 | 25 | 6 | 10 | |
|  | | *Exp* | 1.02 | 4.93 | 0.21 | 0.05 | 0.08 | 37.45 | 0.23 | 17.13 | 9.93 | 10.89 | |
| 400+ | | *Obs* | 0 | 3 | 0 | 0 | 0 | 20 | 0 | 14 | 9 | 2 | |
|  | | *Exp* | 0.62 | 2.45 | 0.06 | 0.01 | 0 | 22.62 | 0.08 | 10.81 | 5.31 | 5.57 | |
| Total deaths in informative strata | |  | 49 | 176 | 113 | 30 | 41 | 1115 | 13 | 447 | 303 | 442 | |
| Score statistic | |  | -1.28 | 0.38 | 0.6 | 1.63 | 0.39 | 0.17 | 1.28 | 2.13 | -0.06 | -1.48 | |
| 1-sided p-value | |  | 0.919 | 0.353 | 0.273 | 0.068 | 0.29 | 0.432 | 0.114 | 0.017 | 0.525 | 0.93 | |
| 2-sided p-value | |  | 0.162 | 0.706 | 0.547 | 0.135 | 0.575 | 0.864 | 0.226 | 0.033 | 0.95 | 0.14 | |
| ERR Sv^-1^ | |  | <-1.933 | 0.446 | 2.745 | 7.002 | 10.571 | 0.072 | 15.736 | 1.489 | -0.036 | -1.106 | |
| 90% CI | |  | (<-1.93, 1.40) | (-0.99, 3.43) | (<-1.93, 16.9) | (<-1.93, 31.5) | (<-1.93, 69.9) | (-0.54, 0.87) | (<-1.93, 78.5) | (0.28, 3.19) | (-0.76, 1.14) | (-1.69, 0.16) | |
| 95% CI | |  | (<-1.93, 2.92) | (-1.15, 4.19) | (<-1.93, 20.6) | (<-1.93, 39.9) | (<-1.93, 87.0) | (-0.63, 1.04) | (<-1.93, 101) | (0.09, 3.57) | (-0.76, 1.43) | (-1.74, 0.48) | |

| **TABLE S3 (cont.) Tests for trend in mortality with dose by cause of death (lagged by 10 years)** | | | | | | | | | | |
| --- | --- | --- | --- | --- | --- | --- | --- | --- | --- | --- |
| Dose (mSv) | | *Number of deaths* | Thyroid | Ill-defined and secondary cancers | Lymphatic or haematopoietic including leukaemia | Non-Hodgkin lymphoma | Hodgkin lymphoma | Multiple myeloma | Malignant neoplasms strongly related to smoking | Chronic Obstructive Pulmonary disease (COPD) |
| <10 | | *Obs* | 16 | 569 | 475 | 186 | 19 | 87 | 2854 | 780 |
|  | | *Exp* | 15.81 | 563.74 | 473.62 | 192.11 | 20.19 | 91.77 | 2895.32 | 744.27 |
| 10- | | *Obs* | 2 | 111 | 102 | 48 | 4 | 20 | 621 | 180 |
|  | | *Exp* | 2.68 | 122.18 | 106.44 | 43.05 | 4.22 | 21.54 | 621.05 | 184.27 |
| 20- | | *Obs* | 4 | 140 | 126 | 51 | 5 | 26 | 793 | 238 |
|  | | *Exp* | 3.91 | 158.12 | 132.98 | 52.75 | 5 | 28.27 | 789.89 | 237.35 |
| 50- | | *Obs* | 2 | 88 | 69 | 21 | 2 | 24 | 470 | 118 |
|  | | *Exp* | 2.31 | 88.15 | 72.07 | 28.51 | 2.17 | 15.23 | 440.33 | 130.27 |
| 100- | | *Obs* | 2 | 81 | 54 | 23 | 4 | 9 | 298 | 82 |
|  | | *Exp* | 1.62 | 57.5 | 47 | 19.17 | 1.28 | 9.89 | 296.29 | 87.39 |
| 200- | | *Obs* | 0 | 30 | 29 | 16 | 0 | 3 | 190 | 43 |
|  | | *Exp* | 1.12 | 32.66 | 26.7 | 11.1 | 0.77 | 5.33 | 180.83 | 52.16 |
| 400+ | | *Obs* | 2 | 23 | 19 | 8 | 0 | 6 | 102 | 28 |
|  | | *Exp* | 0.55 | 19.64 | 15.18 | 6.32 | 0.37 | 2.97 | 104.29 | 33.29 |
| Total deaths in informative strata | |  | 28 | 1042 | 874 | 353 | 34 | 175 | 5328 | 1469 |
| Score statistic | |  | 1.18 | 1.44 | 1.41 | 1.57 | -0.13 | 1.37 | 0.64 | -2.22 |
| 1-sided p-value | |  | 0.124 | 0.075 | 0.08 | 0.058 | 0.5 | 0.086 | 0.26 | 0.987 |
| 2-sided p-value | |  | 0.248 | 0.151 | 0.159 | 0.116 | 0.999 | 0.172 | 0.521 | 0.027 |
| ERR Sv^-1^ | |  | 2.48 | 0.672 | 0.693 | 1.307 | <-1.933 | 1.496 | 0.123 | -0.623 |
| 90% CI | |  | (-0.48, 13.4) | (-0.08, 1.66) | (-0.10, 1.74) | (-0.05, 3.31) | (<-1.93, 20.4) | (-0.21, 4.8) | (-0.18, 0.47) | (-0.96, -0.18) |
| 95% CI | |  | (-0.72, 17.4) | (-0.20, 1.88) | (-0.23, 1.97) | (-0.25, 3.77) | (<-1.93, 26.6) | (-0.42, 5.6) | (-0.23, 0.54) | (-1.02, -0.08) |

| **TABLE S4 Test for trend in cancer incidence with dose (lagged by 10 years)** | | | | | | | | | | | |  |
| --- | --- | --- | --- | --- | --- | --- | --- | --- | --- | --- | --- | --- |
| Dose (mSv) | *Number of cases* | All neoplasms*^1^* | All malignant neoplasms*^1^* | All solid cancers*^1^* | All malignant neoplasms excluding leukaemia*^1^* | All malignant neoplasms excluding lung, pleura and leukaemia*^1^* | Mouth, tongue and pharynx | Oesophagus | Stomach | Large intestine | Rectum | |
| <10 | *Obs^2^* | 12517 | 11270 | 10342 | 10973 | 9033 | 241 | 338 | 435 | 869 | 621 | |
|  | *Exp^3^* | 12714.12 | 11409.47 | 10461.02 | 11116.91 | 9128.87 | 238.33 | 334.52 | 437.8 | 903.87 | 646.02 | |
| 10- | *Obs* | 2454 | 2245 | 2051 | 2192 | 1796 | 48 | 64 | 103 | 208 | 131 | |
|  | *Exp* | 2436.68 | 2234.96 | 2053.77 | 2174.85 | 1746.7 | 38.9 | 64.8 | 101.78 | 188.96 | 122.67 | |
| 20- | *Obs* | 3019 | 2752 | 2526 | 2674 | 2108 | 39 | 82 | 131 | 236 | 150 | |
|  | *Exp* | 3036.25 | 2794.73 | 2572.27 | 2718.61 | 2170.59 | 45.52 | 80.7 | 132.98 | 238.71 | 157.18 | |
| 50- | *Obs* | 1742 | 1606 | 1508 | 1565 | 1222 | 29 | 38 | 84 | 148 | 98 | |
|  | *Exp* | 1649.78 | 1521.15 | 1405.09 | 1480.14 | 1176.11 | 24.7 | 44.73 | 74.97 | 135.24 | 86.7 | |
| 100- | *Obs* | 1131 | 1043 | 963 | 1015 | 794 | 11 | 30 | 45 | 98 | 58 | |
|  | *Exp* | 1068.84 | 986.86 | 913.61 | 960.3 | 758.61 | 15.24 | 30.61 | 50.9 | 91.9 | 54.77 | |
| 200- | *Obs* | 628 | 582 | 534 | 566 | 435 | 6 | 23 | 26 | 45 | 35 | |
|  | *Exp* | 610.96 | 566.47 | 526.51 | 551.13 | 428.46 | 8.54 | 18.67 | 30.25 | 52.75 | 31.24 | |
| 400+ | *Obs* | 351 | 318 | 290 | 311 | 249 | 1 | 9 | 22 | 36 | 22 | |
|  | *Exp* | 325.39 | 302.36 | 281.73 | 294.06 | 227.66 | 3.78 | 9.96 | 17.32 | 28.57 | 16.42 | |
| Total cases in informative strata |  | 21842 | 19816 | 18214 | 19296 | 15637 | 375 | 584 | 846 | 1640 | 1115 | |
| Score statistic |  | 3.15 | 2.48 | 1.950 | 2.55 | 2.27 | -2.08 | 0.08 | 0.47 | 1.21 | 2.17 | |
| 1-sided p-value |  | <0.001 | 0.007 | 0.026 | 0.005 | 0.012 | 0.981 | 0.467 | 0.318 | 0.113 | 0.015 | |
| 2-sided p-value |  | <0.002 | 0.013 | 0.051 | 0.011 | 0.023 | 0.037 | 0.935 | 0.635 | 0.225 | 0.03 | |
| ERR Sv^-1^ |  | 0.332 | 0.271 | 0.221 | 0.283 | 0.278 | -1.524 | 0.048 | 0.2 | 0.423 | 1.084 | |
| 90% CI |  | (0.15, 0.52) | (0.09, 0.47) | (0.03, 0.42) | (0.10, 0.48) | (0.07, 0.5) | (-1.85, -0.44 ) | (-0.76, 1.21) | (-0.42, 1.05) | (-0.13, 1.12) | (0.23, 2.2) | |
| 95% CI |  | (0.12, 0.56) | (0.05, 0.51) | (0.00, 0.46) | (0.06, 0.52) | (0.04, 0.54) | (-1.87, -0.13 ) | (-0.87, 1.48) | (-0.52, 1.24) | (-0.22, 1.28) | (0.09, 2.45) | |

^1^ excludes non-melanoma skin cancer

^2^ the observed number of informative cancer incidence in each cumulative dose category

^3^ the expected number of incidences in each dose category if there were no association between incidence and cumulative dose.

| **TABLE S4 (cont.) Test for trend in cancer incidence with dose (lagged by 10 years)** | | | | | | | | | | | |
| --- | --- | --- | --- | --- | --- | --- | --- | --- | --- | --- | --- |
| Dose (mSv) | *Number of cases* | Liver | Primary Liver | Gallbladder | Pancreas | Larynx | Trachea, bronchus and lung | Pleura | Bone | Connective and soft tissue |  |
| <10 | *Obs* | 126 | 81 | 47 | 284 | 157 | 1741 | 199 | 17 | 68 |  |
|  | *Exp* | 122.44 | 76.04 | 47.73 | 284.45 | 141.41 | 1764.41 | 223.63 | 17.03 | 66.84 |  |
| 10- | *Obs* | 13 | 5 | 14 | 66 | 21 | 347 | 49 | 3 | 11 |  |
|  | *Exp* | 23.81 | 14.43 | 9.89 | 61.96 | 28.14 | 384.67 | 43.48 | 3.48 | 11.26 |  |
| 20- | *Obs* | 33 | 19 | 13 | 71 | 32 | 505 | 61 | 4 | 12 |  |
|  | *Exp* | 28.19 | 17.36 | 12.43 | 78.22 | 35.44 | 490.45 | 57.57 | 3.99 | 13.62 |  |
| 50- | *Obs* | 16 | 11 | 5 | 48 | 12 | 313 | 30 | 3 | 9 |  |
|  | *Exp* | 15.71 | 9.76 | 7.14 | 42.81 | 20.02 | 272.39 | 31.63 | 2.17 | 7.98 |  |
| 100- | *Obs* | 10 | 5 | 2 | 32 | 16 | 186 | 35 | 1 | 9 |  |
|  | *Exp* | 9.06 | 5.59 | 4.74 | 27.64 | 14.34 | 180.54 | 21.14 | 1.28 | 5.46 |  |
| 200- | *Obs* | 7 | 6 | 3 | 10 | 9 | 117 | 14 | 1 | 1 |  |
|  | *Exp* | 4.64 | 3.16 | 2.74 | 16.75 | 9.12 | 110.78 | 11.89 | 0.76 | 3.2 |  |
| 400+ | *Obs* | 1 | 1 | 2 | 10 | 6 | 54 | 8 | 0 | 0 |  |
|  | *Exp* | 2.15 | 1.67 | 1.33 | 9.16 | 4.53 | 59.75 | 6.65 | 0.29 | 1.63 |  |
| Total cases in informative strata |  | 206 | 128 | 86 | 521 | 253 | 3263 | 396 | 29 | 110 |  |
| Score statistic |  | 0.2 | 0.44 | -0.04 | -0.35 | 0.18 | 0.49 | 2.2 | -0.23 | -1.29 |  |
| 1-sided p-value |  | 0.421 | 0.328 | 0.489 | 0.637 | 0.428 | 0.312 | 0.014 | 0.546 | 0.902 |  |
| 2-sided p-value |  | 0.843 | 0.656 | 0.977 | 0.726 | 0.857 | 0.623 | 0.027 | 0.908 | 0.197 |  |
| ERR Sv^-1^ |  | 0.2 | 0.741 | -0.041 | -0.199 | 0.128 | 0.13 | 2.4 | -1.095 | <-1.944 |  |
| 90% CI |  | (-1.50, 3.43 ) | (-1.37, 4.71) | (-1.22, 2.81) | (-0.90, 0.92) | (-0.81, 1.66) | (-0.28, 0.61) | (0.48, 5.22) | (<-1.94, 12.28) | (<-1.94, 0.99) |  |
| 95% CI |  | (-1.65, 4.21 ) | (-1.56, 5.74) | (-1.33, 3.66) | (-1.00, 1.19) | (-0.93, 2.03) | (-0.35, 0.72) | (0.20, 5.88) | (<-1.94, 17) | (<-1.94, 2.02) |  |

| **TABLE S4 (cont.) Test for trend in cancer incidence with dose (lagged by 10 years)** | | | | | | | | | | | |  |  |
| --- | --- | --- | --- | --- | --- | --- | --- | --- | --- | --- | --- | --- | --- |
| Dose (mSv) | | *Number of cases* | Malignant melanoma | Non-melanoma skin cancer | Female breast | Uterus | Ovary | Prostate | Testis | Bladder | Kidney | All Brain | |
| <10 | | *Obs* | 418 | 3112 | 394 | 82 | 49 | 2136 | 129 | 637 | 308 | 346 | |
|  | | *Exp* | 424.99 | 3209.43 | 404.05 | 85.37 | 49.87 | 2148.84 | 123.95 | 638.86 | 307.76 | 346.82 | |
| 10- | | *Obs* | 63 | 608 | 48 | 11 | 5 | 435 | 12 | 134 | 75 | 65 | |
|  | | *Exp* | 65.08 | 592.67 | 43.61 | 9.78 | 5.72 | 428.55 | 16.31 | 136.77 | 62.51 | 60.37 | |
| 20- | | *Obs* | 79 | 737 | 35 | 6 | 5 | 553 | 13 | 157 | 61 | 71 | |
|  | | *Exp* | 74.95 | 748.3 | 29.32 | 6.01 | 3.86 | 564.54 | 16.61 | 173.53 | 78.86 | 68.95 | |
| 50- | | *Obs* | 40 | 452 | 9 | 4 | 1 | 332 | 10 | 102 | 48 | 36 | |
|  | | *Exp* | 39.36 | 411.39 | 7.47 | 1.59 | 0.96 | 307.48 | 7.85 | 93.95 | 42.44 | 37.27 | |
| 100- | | *Obs* | 26 | 282 | 2 | 0 | 0 | 200 | 3 | 61 | 21 | 22 | |
|  | | *Exp* | 24.04 | 268.16 | 3.56 | 0.91 | 0.46 | 196.11 | 3.7 | 60.91 | 26.79 | 23.07 | |
| 200- | | *Obs* | 13 | 172 | 1 | 1 | 0 | 111 | 4 | 42 | 17 | 11 | |
|  | | *Exp* | 11.84 | 147.19 | 0.84 | 0.15 | 0.1 | 106.31 | 1.87 | 34.93 | 14.61 | 12.94 | |
| 400+ | | *Obs* | 7 | 97 | 0 | 0 | 1 | 42 | 0 | 25 | 10 | 5 | |
|  | | *Exp* | 5.74 | 82.87 | 0.14 | 0.18 | 0.04 | 57.17 | 0.71 | 19.05 | 7.03 | 6.58 | |
| Total cases in informative strata | |  | 646 | 5460 | 489 | 104 | 61 | 3809 | 171 | 1158 | 540 | 556 | |
| Score statistic | |  | 0.94 | 3.57 | 0.2 | 0.7 | 2.54 | -1.08 | 0.15 | 2.05 | 0.9 | -0.96 | |
| 1-sided p-value | |  | 0.173 | <0.001 | 0.42 | 0.226 | 0.03 | 0.86 | 0.439 | 0.02 | 0.185 | 0.833 | |
| 2-sided p-value | |  | 0.347 | <0.001 | 0.839 | 0.452 | 0.057 | 0.281 | 0.878 | 0.04 | 0.369 | 0.335 | |
| ERR Sv^-1^ | |  | 0.715 | 0.797 | 0.678 | 5.366 | 8.265 | -0.268 | 0.373 | 0.914 | 0.542 | -0.611 | |
| 90% CI | |  | (-0.43, 2.41) | (0.40, 1.24) | (<-1.94, 7.89) | (<-1.94, 26.2) | (1.12, 34.85) | (-0.62, 0.15) | (<-1.94, 5.61) | (0.16, 1.88) | (-0.37, 1.86) | (-1.28, 0.54) | |
| 95% CI | |  | (-0.59, 2.81) | (0.33, 1.33) | (<-1.94, 9.62) | (<-1.94, 31.9) | (0.63, 43.45) | (-0.68, 0.24) | (<-1.94, 6.97) | (0.03, 2.09) | (-0.50, 2.17) | (-1.36, 0.83) | |

| **TABLE S4 (cont.) Test for trend in cancer incidence with dose (lagged by 10 years)** | | | | | | | |  |  |
| --- | --- | --- | --- | --- | --- | --- | --- | --- | --- |
| Dose (mSv) | | *Number of cases* | Thyroid | Ill-defined and secondary cancers | Lymphatic or haematopoietic including leukaemia | Non-Hodgkin lymphoma | Hodgkin lymphoma | Multiple myeloma | |
| <10 | | *Obs* | 61 | 482 | 933 | 420 | 74 | 142 | |
|  | | *Exp* | 58.89 | 477.5 | 956.57 | 426.69 | 75.45 | 161.87 | |
| 10- | | *Obs* | 10 | 83 | 197 | 90 | 10 | 44 | |
|  | | *Exp* | 8.36 | 102.15 | 182.28 | 80.44 | 10.71 | 31.01 | |
| 20- | | *Obs* | 6 | 131 | 226 | 97 | 15 | 36 | |
|  | | *Exp* | 9.19 | 131.63 | 223.72 | 95.78 | 11.87 | 39.95 | |
| 50- | | *Obs* | 2 | 70 | 101 | 31 | 2 | 27 | |
|  | | *Exp* | 4.97 | 75.18 | 116.76 | 49.16 | 5.81 | 20.79 | |
| 100- | | *Obs* | 5 | 71 | 81 | 33 | 7 | 13 | |
|  | | *Exp* | 2.73 | 51.34 | 73.65 | 30.45 | 3.5 | 13.14 | |
| 200- | | *Obs* | 0 | 29 | 48 | 23 | 2 | 7 | |
|  | | *Exp* | 1.32 | 32.8 | 40.26 | 16.3 | 1.85 | 6.77 | |
| 400+ | | *Obs* | 2 | 24 | 28 | 13 | 0 | 8 | |
|  | | *Exp* | 0.53 | 19.42 | 20.76 | 8.16 | 0.82 | 3.48 | |
| Total cases in informative strata | |  | 86 | 890 | 1614 | 707 | 110 | 277 | |
| Score statistic | |  | 0.95 | 1.44 | 2.27 | 2.12 | -0.16 | 2.59 | |
| 1-sided p-value | |  | 0.164 | 0.075 | 0.012 | 0.017 | 0.535 | 0.005 | |
| 2-sided p-value | |  | 0.329 | 0.15 | 0.023 | 0.034 | 0.929 | 0.01 | |
| ERR Sv^-1^ | |  | 1.437 | 0.686 | 0.919 | 1.261 | -0.588 | 2.806 | |
| 90% CI | |  | (-0.61, 6.61) | (-0.08, 1.71) | (0.22, 1.78) | (0.24, 2.63) | (<-1.94, 6.84) | (0.76, 6.13) | |
| 95% CI | |  | (-0.81, 8.15) | (-0.20, 1.94) | (0.11, 1.97) | (0.08, 2.94) | (<-1.94, 8.92) | (0.48, 6.96) | |
